# Interpersonal Psychotherapy vs. Treatment as Usual for Major Depression Related to Work Stress: A Pilot Randomized Controlled Study

**DOI:** 10.3389/fpsyt.2020.00193

**Published:** 2020-03-18

**Authors:** Elisabeth Schramm, Simon Mack, Nicola Thiel, Carolin Jenkner, Moritz Elsaesser, Thomas Fangmeier

**Affiliations:** ^1^Department of Psychiatry and Psychotherapy, Faculty of Medicine, University Medical Center, University of Freiburg, Freiburg, Germany; ^2^Hochschule Fresenius, University of Applied Sciences, Heidelberg, Germany; ^3^Clinical Trials Unit, Faculty of Medicine, University Medical Center, University of Freiburg, Freiburg, Germany

**Keywords:** interpersonal psychotherapy, work, stress, depression, work intervention, randomized controlled trial

## Abstract

**Background:** Depressive disorders are among the leading causes of sick leave and long-term work incapacity in most modern countries. Work related stress is described by patients as the most common context of depression. It is vital to know what types of treatments are effective in improving work related problems and occupational health. However, there is only limited evidence on work-focused interventions.

**Methods:** The aim of our study was to evaluate the feasibility and generate first data on the effectiveness of Interpersonal Psychotherapy (IPT) adapted as a group program to focus on the work context (W-IPT). In total, 28 outpatients (22 women; *M* = 49.8 years old) with Major Depressive Disorder related to work stress were randomized to 8 weekly group sessions of W-IPT or to treatment as usual (TAU; guideline oriented treatment). Primary endpoint was the Hamilton Rating Scale for Depression (HRSD-24) score. Key secondary endpoints were, among others, Beck Depression Inventory (BDI-II), Work Ability Index (WAI), Return to Work Attitude (RTW-SE), and the Effort-Reward-Imbalance (ERI). In addition, we evaluated the participants' overall satisfaction with the W-IPT program by two items. A follow-up assessment was conducted 3 months after end of acute treatment.

**Results:** W-IPT was significantly more effective than TAU in reducing clinician-assessed depressive symptoms at follow-up (HRSD-24 W-IPT/TAU: *M* = 6.6/12.0, SE: 1.46/2.17, t_(df = 1)_ = −2.24, *p* = 0.035, *d* = 0.79) and self-assessed depression (BDI-II W-IPT/TAU post-treatment: *M* = 8.8/18.8, SE: 1.69/2.70, t_(df = 1)_ = −3.82, *p* = 0.001, *d* = 1.28; follow-up: *M* = 8.8/16.1, SE: 1.62/2.26, t_(df = 1)_ = −2.62, *p* = 0.015, *d* = 0.99). Furthermore, W-IPT was superior in improving work-ability (WAI), return-to-work attitude (RTW-SE), and the effort-reward-ratio (ERI). No dropouts were observed in both groups. The vast majority (89 percent) of participants in the W-IPT condition were “very satisfied” with the program, although wishing for a greater number of sessions (75 percent).

**Conclusions:** A work-focused IPT program for the treatment of depression associated to work stress was feasible and highly acceptable. W-IPT turned out to be more effective than standard treatment in reducing depression and work-related problems. However, further evidence in a multicenter trial extending this pilot study is necessary.

## Introduction

Unipolar depression is highly prevalent at the workplace with every 10th female and every 20th male worker meeting criteria for major depressive disorder (1–3). Among the U.S. workforce, the prevalence of major depressive disorder has been estimated at 7.6% (4). Depressive disorders have a large impact on social and occupational functioning (5) and on the ability to work, represented in days of sick leave and long-term work incapacity. Administrative data from national health statistics documented a threefold increase (208%) in days of sick leave due to mental disorders, particularly depression, between 1997 and 2018 (6). Work related stress has been described as the most common cause of a depression by patients (7, 8). Job strain, low job control, low social support, high psychological demands, effort–reward imbalance, and high job insecurity were confirmed as predictors for common mental disorder, particularly depression (9–14).

Specific treatment of employees suffering from depression has received increasing attention in recent years. In a Cochrane review (15), 23 studies with depressed patients were found examining work-directed and non-work-directed clinical interventions that included sickness absence as an outcome. Non-work-directed, non-specific clinical interventions included antidepressant medication, psychological clinical interventions (cognitive behavioral therapy), usual primary or occupational care, combination of psychological and pharmacological intervention, strengths exercise, and relaxation. Only four work-directed psychological interventions (three cognitive behavioral therapies/CBT with a focus on work, one special care program) added to a non-specific clinical intervention were identified, showing moderate evidence in reducing sickness absence compared to a clinical intervention alone. In contrary, enhancing primary care with a quality improvement program or with physical exercise did not have a considerable effect on work outcomes. The same applied for comparing one antidepressant medication to another. In addition, a systematic meta-review (16) examining the effectiveness of workplace mental health interventions revealed good effects for interventions with a specific focus on work for depression and/or anxiety, such as CBT-based and problem-focused return-to-work programs. Those approaches had a strong evidence base for improving symptomology and a moderate evidence base for improving occupational outcomes. A recent systematic review analyzed the effects of universal and targeted workplace interventions. While targeted interventions are specifically aimed at employees with acute depressive symptoms, universal interventions include employees broadly at all mental health stages and therefore carry out preventive as well as curative effects in a more heterogeneous group. CBT was the most frequently used method, while approaches combining different therapeutic interventions showed the most promising results in reducing depressive symptoms (17). Most treatments were delivered in group format and resulted in lower attrition rates compared with other delivery formats. A group vs. an individual treatment format may increase the subjective experience of finding support from other group members and sharing the similar problematic work-related patterns such as perfectionism and the lack of being able to set limits. The results must be considered with caution, however, since synthesizing data always requires the reduction of information in order to create clear-cut conclusions. Therefore, data is pooled by commonalities potentially omiting distinct features. Nigatu et al. (18) analyzed interventions focusing on enhancing return to work (RTW) in individuals with a common mental illness. The authors concluded that those programs did not lead to improved RTW rates over control conditions, but reduced the number of sick-leave days.

Overall, these findings demonstrate that there are empirically supported workplace directed interventions which facilitate the recovery of employees diagnosed with depression and produced modest effects on occupational outcomes. However, the small number of controlled studies on the effects of psychotherapy on work-related outcomes in MDD to date makes it difficult to draw final conclusions.

The authors of the Cochrane review (15) assert that there is an urgent need to evaluate work-focused treatments by adapting existing psychotherapeutic interventions to focus on the work context and to include work-related outcomes. The Interpersonal Psychotherapy (IPT) is an evidence based first-line treatment for depression (19) recommended in national and international guidelines such as National Institute of Clinical Excellence, the American Psychiatric Association, and the World Health Organization [for summary see (20)]. The effectiveness of IPT in improving depression has been widely demonstrated in numerous controlled trials (21). As IPT directly relates to psychosocial problems associated with the depressive episode, it seems to be an appropriate fit for the treatment of work problems by focusing on work as a social role. Besides the four standard foci of IPT (interpersonal disputes, role transitions, grief, and social deficits), work stress as a fifth focus has been established over the past years (22).

The aim of the present pilot study was to evaluate the effectiveness of IPT tailored specifically to focus on the work context (W-IPT) by additional integrated modules addressing work problems and work stress. Our primary hypothesis was that W-IPT is more effective in reducing depressive symptoms compared to TAU after termination of the 8-weeks program.

## Materials and Methods

### Trial Design

We conducted a monocentric, randomized controlled trial comparing W-IPT vs. TAU in a group format in outpatients with major depression related to work stress between March 2017 and February 2018. The Research Ethics Board of the University of Freiburg approved the trial. In accordance with the Declaration of Helsinki, participants were informed in detail about the purpose and design of the trial, and provided written consent prior to randomization. This trial was registered in advance with the German Clinical Trials Register (registry number: DRKS00011669).

### Participants and Procedure

**Figure 1:**
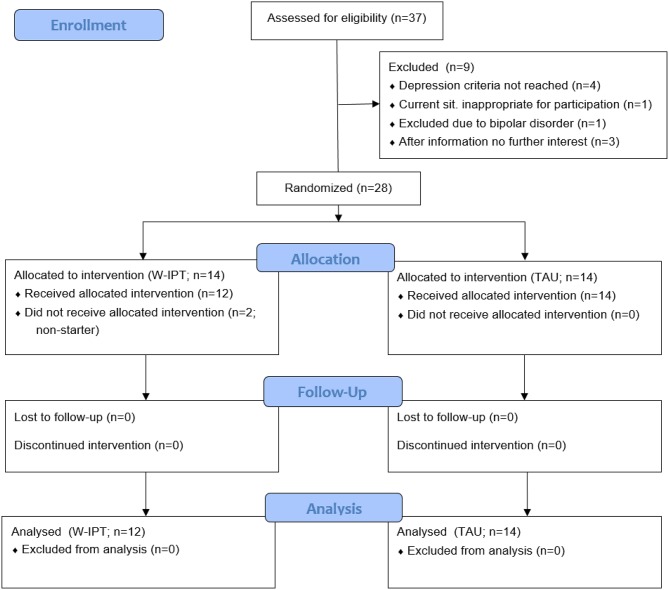
CONSORT flowchart. W-IPT, work-related interpersonal therapy; TAU, treatment-as-usual.

In total, 28 patients from the area of Freiburg, Germany were randomized to W-IPT or TAU. *Randomization* was conducted according to a central computerized randomization schedule (randomizer.at from Medical University Graz, Austria) stratified with the factor medication intake yes/no and a 1:1 treatment allocation ratio. The randomization method was permuted blocks with a block size of two. Eligible patients had a primary diagnosis of major depression (single-episode or recurrent) according to the Structured Clinical Interview for DSM-IV [SCID-I, (23)], and a score of ≥ 20 on the 24-item version of the Hamilton Rating Scale for Depression [HRSD-24, (24)]. Due to recruitment limitations, we allowed to include two patients with a score below 20 on the HRSD-24. The assessments (HRSD-24 and SCID-I) were done by trained clinical psychologists who were blind to the randomization. Eligible patients also fulfilled the criteria for at least one of the following work-related problems: bullying/mobbing, interpersonal conflicts, role transition, role confusion, burnout, boreout, job strain, effort-reward-imbalance, job demand-control imbalance, low decision latitude, high psychological demands, work-life-imbalance, low social support, high job insecurity. All patients were in ongoing medical/psychiatric care, and if pharmacotherapy (antidepressants, no regular use of benzodiazepines) was involved it had to be stable for at least 4 weeks before randomization. Patients were 18–65 years old and fluent in German language. We excluded participants with acute risk of suicide; history of psychotic symptoms, bipolar disorder, or organic brain disorders; a primary diagnosis of another axis I disorder; concurrent diagnosis of substance dependency; antisocial, schizotypal, or borderline personality disorder [SCID-II, (25)]. Figure 1 displays the patient flow.

The *W-IPT* condition followed a guideline (unpublished manuscript). W-IPT focuses on the work context by adding specific elements to the regular IPT strategies, i.e., identifying work-related stress factors using an individual stress- and resource profile; psychoeducation on the association of work stress and depression; creating a balance between performance values and interpersonal values; teaching mindfulness and other coping strategies for work-related stress (including social support); practicing communication skills at work to cope with interpersonal conflicts and difficult role transitions, return-to-work plans, and organization involvement. By applying those strategies, social and interpersonal problems at work contributing to depression are addressed. The intervention includes 1 weekly group session of 90 min for 6–8 outpatients over 8 weeks in addition to TAU. Before the start of the group, one preliminary session was conducted in which the interpersonal inventory and a treatment contract including individual goals were performed. In addition, the patients and therapist decided if it was indicated to involve the employer and/or a social worker. The group sessions were conducted by two out of three professionally trained psychotherapists, certified in IPT treatment.

Patients randomized to *TAU* were encouraged to continue with guideline oriented treatment (including psychotherapy and/or pharmacotherapy) by a primary care physician, psychiatrist, or licensed psychotherapist during the study period. In order to compensate for non-participation in the W-IPT condition, patients in the TAU condition were offered to participate in the W-IPT program after their follow-up evaluation.

### Assessment

All measures were administered at pre-treatment (baseline), posttreatment (8 weeks after baseline), and at follow-up (3 months after posttreatment). Defined pre-specified *primary outcome measure* was the change in the clinician-rated *HRSD-24* (24) from baseline to posttreatment. The HRSD is a clinician-administered clinical interview for the assessment of the severity of depressive symptoms. Scores range from 0 to 54, with higher scores reflecting more depressive symptoms. A score of <9 is qualified as “normal,” 9–16 as “mild,” 17–24 as “moderate,” and a score > 25 as “severe.” Detailed information on psychometric properties of the HRSD-24 can be found elsewhere (26). *Secondary outcome measures* were *remission*, defined as HRSD-24 score of ≤ 8, and *response rate*, defined as the reduction in the HRSD-24 score by at least 50% from baseline; and the self-rated *Beck Depression Inventory-II [BDI-II*, (27)] to assess depressive symptoms from patient's perspective. The test quality criteria, such as internal consistency, validity, and test-retest reliability, are most satisfactory in both clinical and non-clinical subjects [Cronbach's alpha = 0.89, (28)]. For work-related measures, we used the *Work Ability Index [WAI*, (29)], a self-evaluation of the subject's estimated capacities and resources at work. Work ability is considered impaired when the WAI score was low (7–27 points) or moderate (28–36 points), and as adequate when the score was good (37–43 points) or excellent (44–49 points). In addition, we used the long version of the *Effort-Reward Imbalance (ERI)* at work (30). The ERI effort scale consists of three items that refer to demanding aspects at work. The reward scale consists of seven items with an underlying three-factorial structure referring to financial, esteem-related and security-/career-related rewards. Published data document satisfactory internal consistency in terms of Cronbach's α (usually α > 0.70) of the three scales of effort, reward and over-commitment, and a satisfactory test-retest-reliability (31). The *return-to-work self-efficacy scale* [*RTW-SE*, (32)] was applied to assess the subject's belief in the own ability to meet the demands at work. The RTW-SE contains 11 questions on self-efficacy in managing work demands. To evaluate the patient's satisfaction with the W-IPT program, we used 2 self-designed items addressing the overall satisfaction with the content (*5-point-scale ranging from 1* = *very dissatisfied to 5* = *very satisfied*) and with the duration of the program (*rather too short; suitable; rather too long*).

### Sample Size

To obtain estimates for the treatment effect and its variance, the primary outcome clinician-rated *HRSD-24* posttreatment was assessed in 24 patients. Julious (33) found that a sample size of 12 per group in pilot studies seems reasonable for generation of pilot data. To account for potential dropouts, 28 patients were assessed for eligibility.

### Statistical Methods

The primary efficacy analysis was performed according to the intention-to-treat (ITT) principle and therefore was based on the full analysis set (FAS). The FAS included all randomized patients, and patients were analyzed as belonging to their randomized arm regardless of protocol deviations. Patients for which the therapy was not started were excluded. The primary endpoint (change in HRSD-24 score from baseline to 8 weeks of treatment) was analyzed using linear regression with treatment group as factor and baseline HRSD-24 score as covariate. A conservative estimate of the effect size anticipated for the subsequent confirmative trial was derived from these analyses by a combination of clinical and statistical judgement. We analyzed secondary endpoints descriptively in a similar fashion as the primary outcome, using regression models as appropriate for the respective type of data. Treatment effects were calculated with two-sided 95% confidence intervals. No interim analyses of the efficacy endpoint were performed.

## Results

Table 1 presents sample and clinical characteristics of the total study sample (*N* = 28; 79 percent women; average age: 49.8 years). The majority of subjects were women which reflects the distribution of unipolar depression among the genders (2). In both groups, 50 percent were on sick leave at baseline measure. 57.1 percent in the TAU, and 78.6 percent in the W-IPT condition received ongoing psychopharmacological treatment.

**Table 1:** Sample and clinical characteristics at baseline.

	**W-IPT (*****N*** **=** **14)**		**TAU (*****N*** **=** **14)**	
	***N***	***%***	***N***	***%***
Female sex	11	78.6	11	78.6
Ø age (N, M, SD)	14	47.4 (9.8)	14	52.1 (10.8)
Married or cohabiting	9	64.3	8	57.1
Academic degree (post highschool)	5	35.7	5	35.7
Size of business employer				
Small	1	7.1	0	0.0
Middle	3	21.43	1	7.1
Large	9	64.29	13	92.86
Non self-employed	12	85.71	13	92.86
Sick leave	7	50.0	7	50.0
Pharmacotherapy	11	78.57	8	57.1
Mental health problems mainly caused by				
a) working condition^*^	14	81.1	14	77.1
b) private situation	14	18.9	14	22.9

* *(0–100%); patient's perspective*.

### Primary Outcome

**Figure 2:**
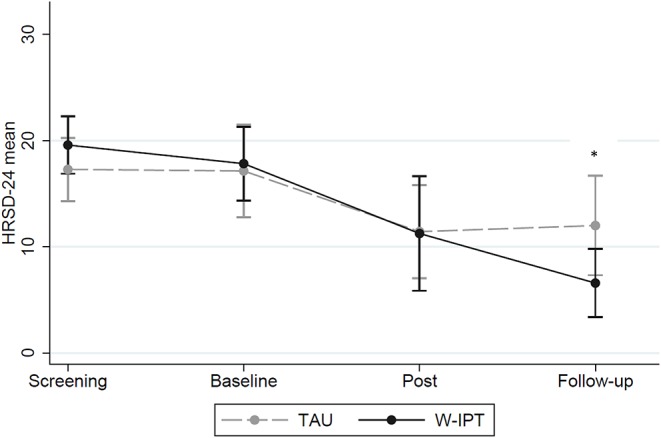
HRSD-24 scores of W-IPT and TAU at screening, baseline, post and follow-up (*N* = 24). *means significant, *p* < 0.05.

Figure 2 shows that there was no significant difference between W-IPT and TAU in reducing clinician-assessed depressive symptoms at post-treatment (*p* = 0.89). However, W-IPT was more effective in improving depressive symptoms than TAU at the follow-up 3 months after the end of the intervention (HRSD-24 W-IPT/TAU: *M* = 6.6/12.0, *SE* = 1.46/2.17, *t*_(df = 1)_ = −2.24, *p* = 0.035). Cohen‘s *d* = 0.79 at follow-up; remission rate 66.7 vs. 35.7%; *p* = 0.24 (Table 2).

**Table 2:** Response- and Remission rates of W-IPT and TAU.

		**W-IPT % (N)**	**TAU % (N)**	***p*^(+)^**	***ES (Cohen's d)***
Response	Post-treatment	50.0 (6)	42.9 (6)	0.716	-
≥50% HRSD-reduction	Follow-up	75.0 (9)	50.0 (7)	0.248	-
Remission	Post-treatment	41.7 (5)	50.0 (7)	0.671	0.02
<9 HRSD-score T0 vs. T2	Follow-up	66.7 (8)	35.7 (5)	0.238	**0.79**

(+) *p-Wert, χ^2^-test or Fisher‘s exact test (n < 5 pro cell). Significant values are bolded*.

### Secondary Outcomes

**Figure 3:**
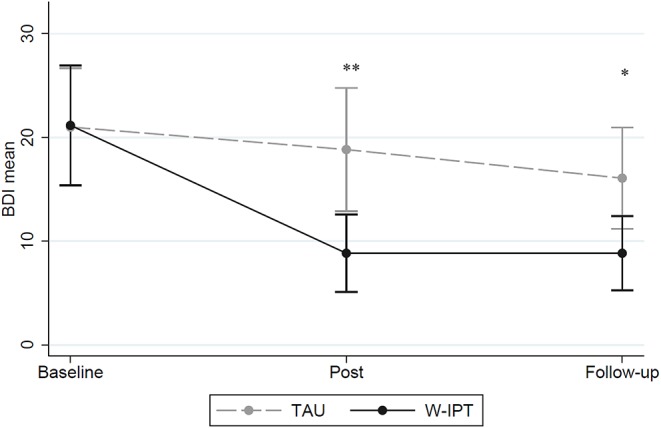
BDI-II scores of W-IPT and TAU at baseline, post and follow-up (*N* = 24). *means significant, *p* < 0.05. **means highly significant, *p* < 0.001.

Figure 3 illustrates the differences of self-rated depression rates between W-IPT and TAU. Group differences were found at posttreatment and follow-up (BDI-II W-IPT/TAU posttreatment: *M* = 8.83/18.83, *SE* = 1.69/2.70, *t*_(df = 1)_ = −3.82, *p* = 0.001; BDI-II follow-up: *M* = 8.83/16.07, *SE* = 1.62/2.26, *t*_(df = 1)_ = −2.62, *p* = 0.015).

**Figure 4:**
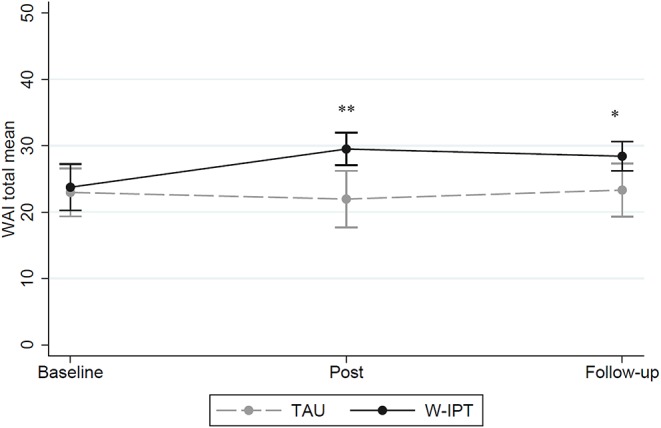
Total WAI mean scores of W-IPT and TAU at baseline, post and follow-up (range: 7–49). *means significant, *p* < 0.05. **means highly significant, *p* < 0.001.

Figure 4 shows the WAI mean scores of W-ITP and TAU at baseline, post, and follow-up (range: 7–49). Group differences were found at posttreatment and follow-up (W-IPT/TAU post-treatment: *M* = 29.5/22.0, *SE* = 1.11/1.97, *t*_(df = 1)_ = 3.41, *p* = 0.002; follow-up: *M* = 27.5/23.3, *SE* = 1.00/1.86, *t*_(df = 1)_ = 2.32, *p* = 0.029).

**Figure 5:**
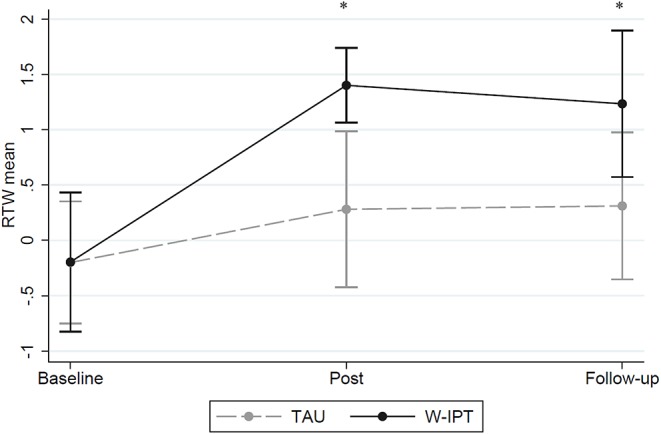
RTW-SE mean scores of W-IPT and TAU at baseline, post and follow-up. *means significant, *p* < 0.05.

Figure 5 shows the RTW-SE mean scores of W-ITP and TAU at baseline, post, and follow-up suggesting significant group differences at posttreatment and follow-up (RTW W-IPT/TAU post-treatment: *M* = 0.3/1.4, *SE* = 0.32/0.15, *t*_(df = 1)_ = −3.20, *p* = 0.002; RTW follow-up: *M* = 0.3/1.2, *SE* = 0.36/0.33, *t*_(df = 1)_ = −1.87, *p* = 0.038).

In Table 3, the effort-reward-imbalance (ERI) subscale scores and the effort-reward-ratio (ERR) are demonstrated for baseline, posttreatment and follow-up. Group differences were identified for reward scores at follow-up, and the effort-reward-ratio differed between groups significantly at post-treatment (ERR W-IPT/TAU: *M* = 1.1/1.7, *SE* = 0.79/1.87, *t*_(df = 1)_ = −3.13, *p* = 0.007).

**Table 3:** Effort-reward-imbalance (ERI) of W-IPT and TAU at baseline, post-treatment, and follow-up.

		**W-IPT**	**TAU**
Effort	T0	20.0	18.9
	T1	18.4	19.5
	T2	18.4	16.6
	T0	24.1	22.3
Reward	T1	27.7	21.4
	T2	^*^27.1	21.7
	T0	20.5	19.1
Overcommittment	T1	17.5	17.2
	T2	16.7	15.1
	T0	1.5	1.5
Effort-Reward-Ratio	T1	^*^1.1	1.7
	T2	1.2	1.3

* *means significant, p < 0.05. Significant values are bolded*.

### Participant's Program Evaluation

The vast majority (89 percent) of participants in the W-IPT condition (N = 12) were “very satisfied” with the program, although wishing for a greater number of sessions (“rather too short”; 75 percent).

## Discussion

The aim of the present study was to evaluate the feasibility and generate first data on the effectiveness of W-IPT compared to TAU for major depression related to work stress. A work directed interpersonal group intervention (W-IPT) was more effective than standard treatment (TAU) in reducing clinician rated as well as self-assessed depression 3 months after the intervention, as well as in improving self-assessed depression at the end of the intervention. In the primary outcome measure, the HRSD-24, we detected a between-group standardized effect size of *d* = 0.79 3 months after treatment. We consider this effect large and clinically meaningful. High response and remission rates were identified for the intervention group with 75 and 66.7%, respectively (vs. 50 and 35.7% in the TAU group) at the naturalistic 3-months follow up. However, those differences did not reach significance, most probably due to the small sample size of this pilot study.

The decrease of depressive symptoms with W-IPT is in line with randomized controlled trials that investigated CBT workplace interventions (16, 17, 34). Compared to a work-related CBT approach (34) including 24 weekly individual sessions which reached an effect size of *d* = 1.63 (pre- to posttreatment), W-IPT showed an effect size of *d* = 0.92 after only 8 weekly group sessions, and *d* = 2.14 at the 3-months follow-up, respectively. In the systematic review of Yunus and others (17), all effect sizes of the 14 targeted interventions at the workplace (mostly CBT or stress management programs) were below the ES reached with the W-IPT program (*d* = 0.79 at 3 months‘ follow-up).

Considering work-related outcomes, W-IPT was superior to TAU in increasing the work ability from a critically poor to a moderate, yet still impaired functioning. The W-IPT group intervention was also superior over TAU in reducing work stress by improving the effort-reward-ratio. Furthermore, the return-to-work attitude was more optimistic in terms of increased self-efficacy-thinking about work in the W-IPT group than in the TAU condition. At present, there is limited evidence of other types of tertiary psychological interventions in depressed patients which evaluated work related outcomes other than RTW rates and sickness absence. One study (35) found that work ability was significantly improved according to WAI (15%) during a 3 years follow-up period after different types of psychodynamic or solution-focused therapy. In our sample, WAI scores of the W-IPT group improved by 20 percent (post-measure) and 17 percent (follow-up).

Another main goal of this pilot study was to determine the feasibility and acceptance of the program by the participants. We observed no dropouts in both groups suggesting a high compliance with the intervention among the patients. In addition, the satisfaction with the group intervention appeared to be very positive. The vast majority of participants in the intervention group (89 percent) were “very satisfied” with the intervention program, although wishing a longer duration of the program (75 percent).

There are several limitations of the present study. First, besides the small pilot sample, patients were heterogenous in their work status. Half of them were on sick leave at the beginning of the study whereas the other half continued working, limiting the generalization of the results. Second, depending on the length of sickness absence, not all applied strategies were a good fit for those patients such as a detailed stepwise plan for RTW for patients who continued working. Third, some of the work related measurements were not applicable for those patients on long-term sick leave. Fourth, we did not systematically assess sickness absence throughout the study. All work outcomes were assessed only through self-report. Fifth, we did not evaluate the medication status throughout the study. Multiple analyses have been performed in this trial. As the primary aim of the study was the assessment of the feasibility of the treatment and to generate pilot data for a confirmatory trial, all results have to be interpreted descriptively and with care. Due to the nature of the data, there was no adjustment for multiple testing. Furthermore, reference is made to the gender and age stratification within each group, but data is not compared along these lines and the small sample number would not allow this. In a future randomized, controlled multicenter trial, the current time of sickness absence will be limited to a maximum of 4 weeks. In addition, we will assess the total time of sickness absence as well as the medication status throughout the study.

To our knowledge, this is the first study comparing a work-related IPT approach with a standard treatment including work related outcomes. The findings provide support for using usual IPT strategies combined with mindfulness techniques focused on the work context. The W-IPT program for the treatment of depression associated to work stress was feasible and highly acceptable. W-IPT turned out to be more effective than treatment-as-usual in reducing depression and work-related problems and the results remained stable over the next 3 months. However, further evidence in a multicenter trial extending this pilot study is necessary.

## Data Availability Statement

The datasets generated for this study are available on request to the corresponding author.

## Ethics Statement

The studies involving human participants were reviewed and approved by the Research Ethics Board of the University of Freiburg. The patients/participants provided their written informed consent to participate in this study.

## Author Contributions

ES and CJ: had full access to all the data in the study and take responsibility for the integrity of the data and the accuracy of the data analysis. ES, NT, TF, and CJ: study concept and design. ES, SM, ME, and TF: acquisition, analysis, or interpretation of data and drafting of the manuscript. SM, CJ, and TF: statistical analysis. ES: obtained funding and administrative, technical, or material support and study supervision. All authors: critical revision of the manuscript for important intellectual content.

### Conflict of Interest

ES received modest book royalties and honoraria for workshops and presentations relating to IPT. The remaining authors declare that the research was conducted in the absence of any commercial or financial relationships that could be construed as a potential conflict of interest.
